# Prescriptions Based upon Diagnosis

**Published:** 1892-03

**Authors:** E. V. Ross

**Affiliations:** Rochester, N. Y.


### PRESCRIPTIONS BASED UPON DIAGNOSIS.

Editor of The Homœopathic Physician:—Through the kindness of Dr. Hermance, I am permitted to present the report of the following case of traumatism to the readers of your valued journal, thinking that it may be of some interest. We see that the Doctor treated his patient, and the result was all that could be desired, while Dr. Nisbet, a representative of that body in the medical profession who unjustly lays claim to everything that pertains to science in medicine, would base his treatment upon his diagnosis, which, if wrong (and it undoubtedly was), it would necessarily follow that the treatment would be likewise. This is one of the dangers of old school methods, and yet it is held up before the world as being the *scientific* method.

I do not wish to be understood as ignoring diagnosis or pathology. Hahnemann never discouraged this; he simply tells us to keep in view the image of the sickness which leads to these results, and warns against prescribing upon a pathological hypothesis.

Rochester, N. Y. E. V. Ross.

---

[The case above referred to, and which may be found upon the next page, is an excellent illustration of the necessity of prescribing according to the symptomatology and not the diagnosis. —Ed.]

